# Intestinal Helminth Communities of Grey Partridge *Perdix perdix* and Common Pheasant *Phasianus colchicus* in Poland

**DOI:** 10.3390/ani11123396

**Published:** 2021-11-28

**Authors:** Izabella Rząd, Agata Stapf, Sławomir Adam Kornaś, Ewa Dzika, Rusłan Sałamatin, Adam Kaczmarek, Jerzy Kowal, Marek Wajdzik, Kazimierz Zalewski

**Affiliations:** 1 Institute of Marine and Environmental Sciences, University of Szczecin, ul. Wąska 13, 71-415 Szczecin, Poland; 2 Molecular Biology and Biotechnology Centre, University of Szczecin, ul. Wąska 13, 71-415 Szczecin, Poland; 3 Department of Biological Sciences, Faculty of Sport Science in Gorzów Wielkopolski, Poznan University of Physical Education, ul. Estkowskiego 13, 66-400 Gorzów Wielkopolski, Poland; a.stapf@awf-gorzow.edu.pl; 4 Department of Zoology and Animal Welfare, Faculty of Animal Science, University of Agriculture, Al. Mickiewicza 24/28, 31-059 Kraków, Poland; slawomir.kornas@urk.edu.pl (S.A.K.); jerzy.kowal@urk.edu.pl (J.K.); 5 Department of Medical Biology, Collegium Medicum, University of Warmia and Mazury in Olsztyn, ul. Żołnierska 14 c, 10-561 Olsztyn, Poland; e.dzika@uwm.edu.pl; 6 Department of General Biology and Parasitology, Medical University of Warsaw, ul. Chałubińskiego 5, 02-004 Warsaw, Poland; ruslan.salamatin@wum.edu.pl; 7 Faculty of Medicine, Collegium Medicum, Cardinal Stefan Wyszynski University in Warsaw, ul. Kazimierza Wóycickiego 1/3, 01-938 Warsaw, Poland; adam.kaczmarek@uksw.edu.pl; 8 Department of Forest Biodiversity, Faculty of Forestry, University of Agriculture, Al. 29-Listopada 46, 31-425 Kraków, Poland; marek.wajdzik@urk.edu.pl; 9 Department of Biochemistry, Faculty of Biology and Biotechnology, University of Warmia and Mazury in Olsztyn, ul. Oczapowskiego 1a, 10-719 Olsztyn, Poland; k.zalewski@uwm.edu.pl

**Keywords:** parasites, grey partridge, common pheasant, nematodes, *Capillaria phasianina*, *Heterakis gallinarum*, *Raillietina friedbergeri*, *Brachylaima* sp.

## Abstract

**Simple Summary:**

The presence of intestinal parasites such as nematodes, cestodes, and trematodes is a serious problem for programmes for the conservation of partridges and pheasants, mainly involving the breeding of these birds and their release into the natural environment. These parasites can cause disease in these birds, whether farmed or free-living. The aim of this study was to describe the morphology of parasitic worms in the partridge, native to Poland, and the introduced pheasant, and to determine the level of infection of these birds with intestinal parasitic worms. The study showed that partridges are infected with several helminth species that had not previously been recorded in this species in Poland. Pheasants are more often infected by intestinal nematodes than are partridges. These worms can negatively affect the condition of partridges and increase their risk of infection with pathogenic protozoa. The results of parasitological examination should be used to develop programmes for diagnosis and monitoring of parasitic infections in order to keep flocks free of parasites.

**Abstract:**

The aim of this study was to describe the morphology and means of identification of helminths in native partridges (65) and introduced pheasants (32) in Poland and to determine the level of intestinal infection of these birds by helminths using parasitological and ecological indices. The birds were acquired during the hunting season in the years 2015–2017. Nematodes, *Capillaria phasianina*, cestodes, *Railietina friedbergeri*, and one trematode, *Brachylaima* sp. were recorded for the first time in partridges in Poland. Our findings indicate that parasites are more prevalent in pheasants (prevalence 70.4%) than in partridges (prevalence 50.0%). The component community and infracommunity of parasites of partridges are more diverse (Simpson’s diversity index: 0.63 and mean Brillouin diversity index: 0.10 ± 0.17) and less dominated by a single parasite species (*Capillaria* sp., Berger-Parker dominance index: 0.53) than the pheasant parasite community (Simpson’s diversity index: 0.07, mean Brillouin diversity index: 0.005 ± 0.02, dominant species *Heterakis gallinarum*, Berger-Parker dominance index: 0.96). There were statistically significant differences between partridges and pheasants in the Brillouin diversity index and in the prevalence of *Heterakis gallinarum* (55.6% in pheasants vs. 19.0 in partridges). There were significant differences between wild and farmed partridges in the prevalence of infection by *Capillaria* sp. (4.3% vs. 37.5%) and *H. gallinarum* (39.1 vs. 6.2%). In conclusion, the pheasant was shown to be a reservoir, carrier, and shedder of nematodes, which may increase the risk of infection in partridges.

## 1. Introduction

Parasites of the partridge and the pheasant are well known in Europe and Poland [1,2,3,4,5,6,7]. However, no studies have been conducted in Poland to determine whether there is a link between the parasitic fauna of partridges and pheasants or to investigate the role of parasites in changes in the population size of these birds. Faunistic studies of the helminths of partridges and pheasants in Poland were conducted in the 1980s and 1990s [8,9,10], but the current rate of infection is unknown. The only recent research was a study on ornamental pheasants conducted in the period 2015–2018, which showed the presence of a new species of nematode, *Heterakis isolonche*, Linstow, 1906 [11]. This prompted us to undertake parasitological research on the partridge and pheasant in Poland and to attempt to assess the influence of parasites on the health, condition, and population size of these birds. Such studies are already carried out in other European countries, mainly with respect to the role of parasites among various factors contributing to the decline in partridge populations [7,12,13,14,15,16].

Due to programmes to breed and introduce these birds to nature, their condition and health, including parasite infection status, should be tested regularly. According to Tompkins et al. [12,13], partridges and pheasants have a common group of nematodes that parasitize the digestive tract. One species in this group is *Heterakis gallinarum* (Schrank, 1788). The authors argue that these roundworms are transmitted to partridges in habitats previously occupied by pheasants. Infection with *H. gallinarum* impairs the physical condition of partridges and exposes them to the risk of infection with pathogenic protozoa [12]. *H. gallinarum* can be a carrier of the protozoon *Histomonas meleagridis*, which can cause high mortality rates in partridges, whereas pheasants are less susceptible to infection by this parasite [5,15]. An analysis by Potts [15] of long-term changes in the prevalence of nematodes and of histomoniasis indicated strong correlations between them in young and adult partridges and in young pheasants, but not in adult pheasants. Due to differences in the pathogenicity of *H. gallinarum* and *H. isolonche* for partridges and pheasants, species identification of these nematodes based on morphology and/or molecular analysis is an important element of parasitological research.

The grey partridge and common pheasant are game birds, valued by hunters due to long hunting traditions, which has led to the introduction of programmes for their conservation, mainly through breeding in aviaries and release of reared birds into the wild [17,18]. The most important factors determining trends in numbers of partridges are known, owing to analysis of research conducted in the United Kingdom and other European countries, which was summed up by Kuijper et al. and Tapper et al. [14,19]. Analysis of the decline in partridge populations indicates multiple causes: a sharp decrease in the survival rate of chicks due to changes in foraging conditions resulting from the use of pesticides, deteriorating habitat quality due to intensification and mechanization of agriculture, a decrease in the number of available breeding sites, reduced breeding success, and an increase in the role of predators [14]. The present parasitological study was prompted by a marked decline in the number of partridges observed in the last 30 years, both in Poland and in Europe as a whole [14]. A long-term study by Panek [20] showed a decrease in average brood size and in chick survival (by over 20%), probably due to the increase in pesticide use. 

The common pheasant is considered an invasive species. It was purposely introduced to Europe from Asia, mainly China, during the Middle Ages or even earlier, to be bred for hunting and culinary purposes [21]. Its habitats are found in shaded spots in agricultural areas [22]. Until recently, its numbers were falling, as in the case of the partridge, but since the 1990s its population in Poland has been growing. A marked increase in its numbers was noted in the southwest of in Poland, where the mild climate is conducive to its development [23].

The aim of this study was to describe the morphology and means of identifying of helminths in partridges and pheasants in Poland and to determine the level of intestinal infection of these birds by helminths using parasitological and ecological indices. Our main research hypothesis is that parasite load may be the cause of the decline in partridge populations, while pheasants may be a reservoir of parasites in the environment. This is suggested by the occurrence of the same species of parasites in both species of birds and the documented harmfulness of certain parasitic species for their hosts.

## 2. Material and Methods

A parasitological analysis was performed on the intestines of 65 partridges and 32 pheasants from Poland, from the Kuyavian-Pomeranian, Podlaskie, Silesian, and Świętokrzyskie Provinces. The birds were acquired in the period 2015–2017 from hunters during the hunting season (September to February). Hunting took place in compliance with the Game Law of 13 October 1995 (Journal of Laws 1995.147.713, as amended) and the Regulation of the Minister of the Environment of 23 March 2005 on detailed conditions for hunting and identification of carcasses (Journal of Laws 2005.61.48, as amended), as well as the Regulation of the Minister of the Environment of 16 March 2005 on specification of hunting seasons for game animals (Journal of Laws 2005.48.459). Free-living partridges were born in the wild, while farmed partridges were reared in an aviary and released into the wild in August (before the hunting season). Farm-reared partridges were marked. All pheasants were farm-reared and were not marked (Table 1).

Only carcasses of birds in good condition were examined (their numbers are given in Table 1); birds whose intestines were destroyed by gunshots were excluded (their numbers are not taken into account in the study).

The contents of the intestine, together with the scraped intestinal mucosa, were rinsed under running water and examined for the presence of parasites under a stereo microscope (5–40×). Helminths were washed with distilled water and then fixed in 75% ethyl alcohol. Microscope slides were prepared, and morphological observations and morphometric measurements were performed using biological light microscopes (10–400×) [24,25]. Helminths were identified to species on the basis of morphological and morphometric characterization of specimens, using standard taxonomic references [26,27,28,29,30] and original works [4,31,32,33,34,35,36,37]. In the case of parasite specimens that were mechanically damaged when the digestive tract was opened with scissors and or when the mucosa was scraped, the morphological characters necessary for species identification were not visible. In this case, the specimens were identified to the genus level (see *Capillaria* sp.).

Ecological indices describing the component community and infracommunity of parasites in both the partridge and the pheasant were calculated: (1) Simpson’s diversity index, (2) the Berger-Parker dominance index, specifying the dominant species in the component community of parasites, and (3) the Brillouin biodiversity index for the infracommunity of parasites [38]. The component community is the set of all infrapopulations of parasites associated with some subset of a host species; in our case, this was the set of all helminths in the partridge population and helminths in the pheasant population. The infracommunity is the set of parasite infrapopulations in a single host, i.e., in our study the community of helminths in a single host. The infrapopulation includes all individuals of a species in an individual host [39]. Past v.2.11 software was used to calculate the indices [40]. The differences in the Brillouin index between the infracommunities of partridges and pheasants were analysed using the Mann-Whitney U test in STATISTICA software v. 13.1 (StatSoft Polska, Krakow, Poland). Parasitological indices, i.e., prevalence, mean intensity of infection, and range of intensity, were calculated [39]. Prevalence was expressed as the percentage of partridges and pheasants infected with helminths among all those tested in a given province. Mean intensity of infection was determined by calculating the average number of parasites per infected bird. The range of intensity was defined by the minimum and maximum number of parasites per host [39]. The prevalence and intensity of infection of partridges and pheasants were compared, along with the prevalence and intensity of infection of free-living partridges with those indices in farmed partridges. Ecological and parasitological indices were calculated separated for each province. Statistical differences between prevalence of partridge parasites and pheasant parasites and between parasite prevalence in free-living and farm-reared partridges were analysed using the chi-square test. When the assumptions of the test were not met, i.e., the values were less than 5, Fisher’s exact test was used. Differences between the intensity of infection of partridges and pheasants and between intensity of infection of free-living and farmed partridges were analysed using the non-parametric Mann-Whitney U test. The significance level adopted in the statistical analyses was *p* ≤ 0.05. STATISTICA 13.1 software was used for the calculations. 

## 3. Results

### 3.1. Taxonomic Classification of Parasites

The following helminth taxa were recorded in partridges: one cestode, *Raillietina friedbergeri* (Linstow, 1878) (Cestoda, Davaineidae); one trematode, *Brachylaima* sp. (Digenea, Brachylaimidae); and nematodes, *Capillaria phasianina* (Kotlán, 1940) (Nematoda, Capillariidae), *Heterakis gallinarum* (Schrank, 1788) (Nematoda, Heterakidae), and *Capillaria* sp. In the pheasant, two nematode species, *Capillaria phasianina* and *Heterakis gallinarum*, as well as *Capillaria* sp., were recorded. The morphological and morphometric traits of nematodes, which were the largest group of helminths, and cestodes, which were much less numerous, are presented below. There was only one trematode specimen, so it is not possible to describe individual variation in morphology or the ranges of measurements of the body and internal organs. 

#### 3.1.1. *Capillaria* (*Capillaria*) *phasianina* (Kotlán, 1940)

Morphometric measurements are presented in Table 2. 

**Figure 1:**
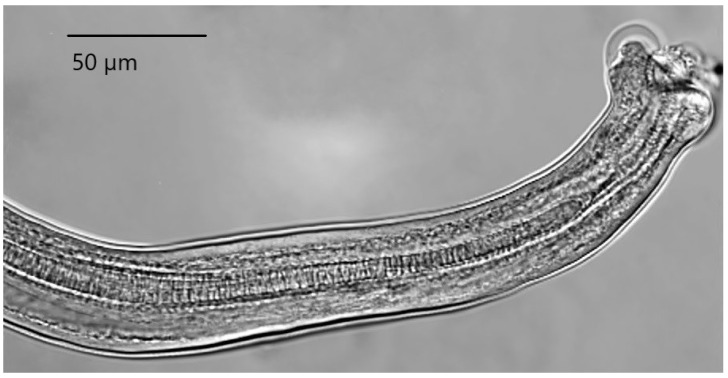
Spicule of male *Capillaria phasianina*; ventral view.

The body of these nematodes is hair-like in shape and milky-white. The caudal end of the body of males is enlarged, with two well-formed lobes. The caudal alae are highly reduced, which is clearly visible on the ventral side. There is a single, well sclerotized spicule (Figure 1).

**Figure 2:**
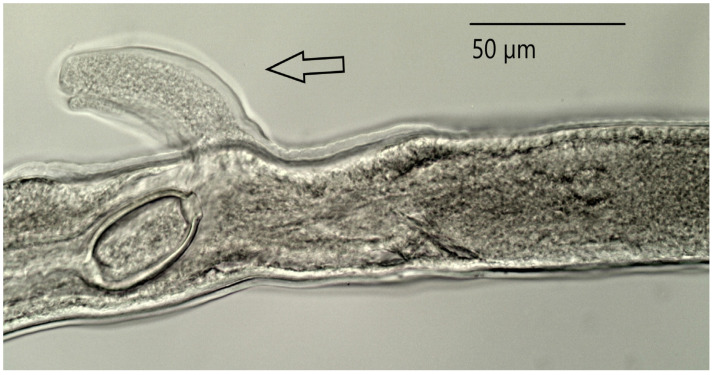
Vulvar area in female *Capillaria phasianina.* The arrow indicates the tubular appendage.

The proximal end of the spicule, which broadens into a knob or funnel shape, is highly characteristic of the genus. The distal end of the spicule is narrow and rounded. The entire length of the copulatory spicule is located in the spicular sheath, whose distal end is covered with spines. At the end of the vulva there is a characteristic tubular appendage (Figure 2).

The eggs are elongated, with a thick shell with visible protruding polar plugs. They are jar- or barrel-shaped. The end of the female body is a rounded tail.

#### 3.1.2. *Heterakis gallinarum* (Schrank, 1788)

**Figure 3:**
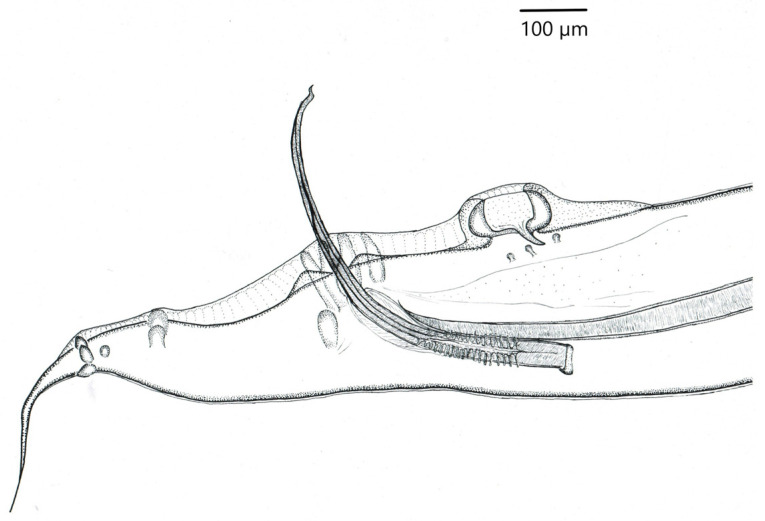
Posterior end of body of male *Heterakis gallinarum*; lateral view.

The nematodes are small with a white body. The males reach 6.65–10.87 mm in length, and the females 9.25–12.6 mm. Average body width, measured at the site of the pear-shaped widening of the oesophagus in individuals of both sexes, is 0.288 mm in males and 0.339 mm in females. The nematodes in the material have typical morphological characteristics for the species. On the posterior end of the body of males there is a copulatory bursa with two well-formed alae. At a distance of 0.576–0.618 mm from the end of the body there is a single pseudosucker with thick, heavily chitinized walls. On the ventral side, 12 pairs of caudal papillae are visible. There are two spicules of very different lengths. The right spicule is 1.936–2.194 mm long, with a sharply pointed end. The shorter left spicule is 0.64–0.765 mm, with a hook-shaped distal end, and is surrounded by two elongated alae (Figure 3).

In females, the vulva is indistinct and located 4.306–6.40 mm from the anterior end of the body. At the posterior end of the body of females there is a straight, gradually narrowing tail. The ellipsoidal eggs, with an average length of 0.06 mm and an average width of 0.04 mm, have thick, smooth shells.

#### 3.1.3. *Raillietina friedbergeri* (von Linstow, 1878)

**Figure 4:**
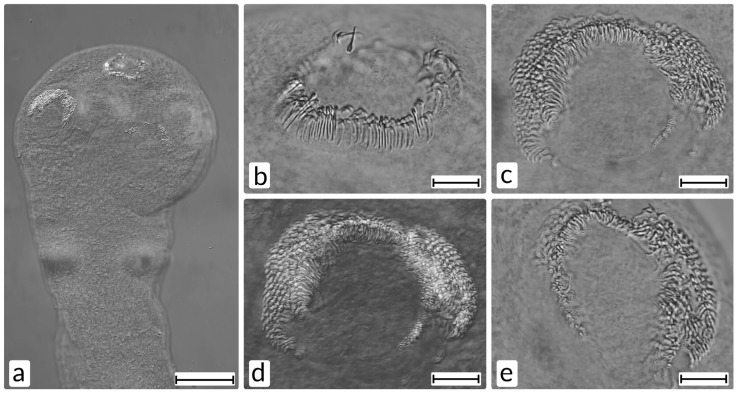
*Raillietina friedbergeri*; (**a)**, scolex; (**b**), armature of rostellum; (**c**–**e**), armature of suckers. Scale bar: a, 100 μm, (**b**–**e**), 20 μm.

Scolex, 0.300 mm long and 0.35 mm wide. Oval suckers, 0.075 × 0.100 mm, with several rows of small hooks, 0.010 mm in length. Rostellum small, diameter 0.70 mm, with a double ring of hooks 0.012 mm in length (Figure 4).

### 3.2. Prevalence, Intensity and Community Characterization Indices

Twenty nine of 58 partridges (50%) and 19 of 27 pheasants (70%) were infected with helminths (Table 3).

No parasites were recorded in birds from the Kuyavian-Pomeranian or Podlaskie Province. The component community of helminths is more biodiverse, as expressed by the Simpson index, in the partridge (0.626) than in the pheasant (0.070). The Berger-Parker dominance index is higher in the helminth community of the pheasant (0.964) than in that of the partridge (0.535), and the dominant species, i.e., the most common, were *H. gallinarum* in the pheasant and *Capillaria* sp. in the partridge. The infracommunity of helminths of partridges is more biodiverse, as expressed by the Brillouin index (0.10 ± 0.17), than that of pheasants (0.005 ± 0.02), and the difference was statistically significant (Mann-Whitney test, *p* = 0.044).

The highest prevalence (nearly 56%), as well as the highest intensity of infection (on average 21.3 in a range from 1 to 191), was recorded for the nematode *H. gallinarum* in the pheasant. The difference between the prevalence of *H. gallinarum* in the partridge and in the pheasant is statistically significant (chi-square test = 11.617, *p* = 0.00065), while the remaining differences in prevalence and intensity of infection between the helminth community of the partridge and the pheasant are not statistically significant.

### 3.3. Differences in Helminth Infection between Wild and Farmed Partridges 

The prevalence of infection with helminths in free-living and farm-reared partridges was similar: 47.8% in free-living partridges (11 birds) and 43.7% in farmed partridges (7 birds). The difference between the prevalence of *Capillaria* sp. in free-living and farmed partridges (4.35%—1 bird vs. 37.5%—6 birds) was statistically significant (chi-square test; Fisher’s exact, *p* = 0.0127). The difference between the prevalence of *H. gallinarum* in free-living and farmed partridges (39.1%—9 birds vs. 6.25%—1 bird) (chi-square test, Fisher’s exact, *p* = 0.0279) was also statistically significant. The differences between free-living and farmed partridges in the prevalence of *C. phasianina* (13.0%—3 birds vs. 12.5%—2 birds), *R. friedbergeri* (0.0% vs. 6.25%—1 bird), and *Brachylaima* sp. (0.0% vs. 6.25%—1 bird) were not statistically significant.

The mean intensity of infection by helminths was higher in farm-reared birds than in free-living birds (20.6 vs. 7.5), but the difference is not statistically significant. The difference between intensity of infection by *C. phasianina* in wild and farmed birds was not statistically significant. The remaining differences between intensity of infection by *Capillaria* sp. and *H. gallinarum* could not be determined due to the low prevalence of these parasites (single cases of infection by *Capillaria* sp. in free-living birds and by *H. gallinarum* in farmed birds).

## 4. Discussion

The aim of the study, which was to describe the morphology and means of identification of helminths of partridges and pheasants in Poland and to determine the level of intestinal infection of these birds by helminths using parasitological and ecological indices, was achieved. This was an important goal because some species of parasites can be present in both species of birds, causing disease symptoms posing a threat to their health and condition and thus potentially affecting the population size of both the partridge and the pheasant. Our hypothesis that parasite load can contribute to a reduction in the partridge population is supported by the presence in the partridges of nematodes of the genus *Capillaria* and the species *Heterakis gallinarum*, which are pathogenic for partridges. We also confirmed that pheasants can be a reservoir of parasites of the genus *Capillaria* and the species *Heterakis gallinarum* in the environment.

### 4.1. Taxonomic Identification of Parasites

Description of the means of identifying parasites was an important objective of the research, as correct identification of nematodes of the genera *Capillaria* and *Heterakis* to the species level is not a simple task. Our presentation of the details of the structure of *Capillaria phasianina* provides data that can be used in future analyses of individual variation within the species. Although *H. gallinarum* is a well-known parasite, the description of its morphology is important, as the detailed data confirm that the specimen was in fact of this species and not of the similar but morphologically different *Heterakis isolonche*, which was recently found in Poland [11]. The morphological traits observed in nematodes of the genus *Capillaria* in our material are in agreement with descriptions and images of the species *C. phasianina* presented by Madsen [33], Baruš and Sergejevej [32], Kellogg and Prestwood [41], and Tampieri et al. [4]. Characteristic traits of representatives of the species *Capillaria phasianina* are the presence of a barrel-shaped appendage next to the vulva in the female, and a caudal segment ending in a pseudobursa with extremely small caudal alae in the male. The morphological traits observed in nematodes of the genus *Heterakis* (Dujardin, 1845) are in agreement with descriptions and images of the species *H. gallinarum* presented by Baruš et al. [32], Park and Shin [42], and Gürler et al. [43]. Morphological characteristics of diagnostic importance for the species *H. gallinarum* are the presence of three labia surrounding the mouth, a pharynx with a pear-shaped extension, two spicules of different lengths, and a single large pseudosucker in the males. Morphometric measurements of *H. gallinarum* individuals from the present study are similar to those presented in works by other authors and are within their ranges [32,42,43]. Only the results of research by Sheikh et al. [44] suggest that the authors of that study incorrectly identified the species of these specimens. Most of the measurements were heavily averaged, without regard to the individuals’ sex. The authors of that study also presented a morphological description specifying that there were two spicules of equal length in males. This calls into question the identification of nematodes by Sheikh et al. [44] as *H. gallinarum*.

The morphological and morphometric traits of the tapeworm *Raillietina friedbergeri* are typical for the species and in agreement with descriptions presented in original works by Linstow [35] and Kornyushin [34]. The single trematode, identified to genus level as *Brachylaima*, is typical for the genus decription presented by Pojmanska (Gibson et al. [26]).

### 4.2. Species Richness of Helminths and Parasitological and Ecological Indices of Infection 

Nematodes, *Capillaria phasianina*, cestodes, *Railietina friedbergeri*, and one trematode, *Brachylaima* sp., were recorded for the first time in partridges in Poland. Our findings indicate that parasites are more prevalent in pheasants than in partridges. The component community and infracommunity of parasites of partridges are more diverse and less dominated by a single parasite species than the pheasant parasite community. Literature data, described below, on the more frequent occurrence of *Capillaria* nematodes in the partridge and of *H. gallinarum* in the pheasant are confirmed in our research material. We focused primarily on the occurrence of nematodes, because they can be pathogenic for birds, and they were the most prevalent and most numerous group of parasites. Tapeworms were much less common, and the literature contains no information indicating that they are pathogenic. The effect of flukes *Brachylaima* sp. on the partridge population is difficult to define, as we recorded only a single specimen. 

The earliest data on helminths of partridges in Poland date back to the 1950s and 1960s. The following nematodes parasitizing the intestines of these birds have been recorded: *Heterakis gallinarum*, *Ascaridia compar* (Schrank, 1790), *A. galli* (Schrank, 1788), *Trichostrongylus tenuis* (Mehlis, 1846), and four species belonging to the genus *Capillaria*: *C. caudinflata* [45,46,47], *C. bursata* (Freitas & Almeida, 1934) [45], and *C. obsignata* and *C. collaris* (Linstow, 1873) [46]. The most numerous are nematodes of the genus *Capillaria* and of the species *H. gallinarum* [33,46,47]. The life cycle of the nematodes *C. phasianina* includes paratenic hosts (Oligochaeta) [6]. The development of the invasive larva in the egg is temperature-dependent and lasts from 23 to 54 days. The typical definitive host is the pheasant *P. colchicus*, in which the larvae reach sexual maturity 25 days after infection [32,48]. The nematode *C. phasianina* was previously recorded in partridges in Denmark and Great Britain [41]. *C. phasianina* is a common species in pheasants, with much lower prevalence in partridges [5,32]. Madsen [33] reported the occurrence of nematodes of the species *Capillaria cadovulvata* in these hosts, but the author’s later work, comparing the morphology of *C. phasianina* described by Kotlan with *C. cadovulvata*, stated that the species are synonymous [33]. According to Kellogg and Prestwood [41], the two researchers described the species independently, and the authors also cites results published by Clapham [5] as confirmation of the occurrence of *C. phasianina* in *Perdix perdix*. 

*Heterakis gallinarum* (Schrank, 1788) is a cosmopolitan species. The typical host of this nematode is the pheasant *P. colchicus*. It is very common in birds of the order Galliformes but has also been found in hosts representing the Anseriformes and Passeriformes [45]. The nematode *H. gallinarum* has a simple development cycle, which may involve Oligochaeta as a paratenic host [6]. The definitive host becomes infected by consuming eggs containing invasive larvae with its food. Nematodes inhabit the caecum, where they reach sexual maturity after 27 days [32]. Although the isolated nematodes represented species whose typical hosts are pheasants, their occurrence in wild partridges is not surprising. Acquisition of nematodes of the genera *Capillaria* and *Heterakis* is influenced by the type of food consumed by partridges and by contact with pheasants in nature. Pheasants have been shown to be a reservoir of *H. gallinarum*, and prevalence of nematodes of this species is higher in these birds than in partridges. The reverse relationship can be inferred from the high prevalence of nematodes of the genus *Capillaria* [45]. Tompkins et al. [12] noted a prevalence of *Capillaria annulata* as high as 83.33% in partridges and only 58.33% in pheasants. Therefore, we should expect a higher prevalence of nematodes of the genus *Capillaria* than of the species *H. gallinarum* in partridges, and the reverse in pheasants. This is confirmed by the present study and by Kozakiewicz et al. [47], where the prevalence of the genus *Capillaria* in 74 partridges shot by hunters reached 16.2% and that of *H. gallinarum* was 2.7%. The same authors emphasized that capillariasis is one of the main invasive diseases of partridges. As mentioned above, the reverse relationship should be found in the nematode fauna of pheasants. A higher prevalence of *H. gallinarum* than of *C. phasianina* in pheasants was reported by Okulewicz and Modrzejewska [49], i.e., 76.7% and 26.7%, respectively, as well as by Tampieri et al. [4]: 37.25% and 35.3%. Ewald and Touyèras [50] emphasize that intensification of agriculture has caused a decrease in the number of partridges in England, and that *H. gallinarum* spread by pheasants may be partly responsible for the decline. 

The results of the present study and the lack of literature data on the structure of helminth communities of partridges and pheasants suggest the need to continue and expand the research. Due to the relatively high prevalence of *H. gallinarum* in comparison to the results of earlier studies, it can potentially pose a health threat to populations of both free-living and farmed birds of the order Galliformes. Tompkins et al. [12] conducted experimental research and a study using a model on the potential effect of *H. gallinarum* on partridge exclusion in the UK. The authors showed that when the pheasant is present in the model, the partridge population is affected by parasites of the species *H. gallinarum*, which are common to both species of birds, but if the pheasant is not present in the model, *H. gallinarum* disappears from the system [13]. The experiments confirmed earlier suppositions that the decline in the partridge population in the UK in the last 50 years may partly be due to apparent competition with pheasants [13]. According to Tompkins et al. [13], separation of the habitats of the partridge and the pheasant would be helpful, but not sufficient, for the survival of the partridge. Research by Sage et al. [16], however, does not confirm the hypothesis that *H. gallinarum* maintained in the habitat by pheasants can negatively affect the productivity and survival of the partridge. The authors point out that unknown circumstances may contribute to such a negative effect of infection of partridges by *H. gallinarum* in nature [16]. Kozakiewicz et al. [47] noted a low prevalence of *H. gallinarum*, but due to infection with other parasites, they drew attention to the need for systematic diagnosis of infections of partridges by parasites. 

The references cited above indicate that the effect of *H. gallinarum* on the survival of partridges is unclear. There is no doubt, however, that the presence of pheasants and partridges in a common habitat is conducive to the development of the population of *H. gallinarum*, contributing to an increase in the prevalence and intensity of infection not only in pheasants but also in partridges. At the same time, the high prevalence of *H. gallinarum* can have a potential negative effect on the biodiversity of the component community and infracommunity of parasites of partridges.

Cestodes previously recorded in partridges in Poland are *Railietina cesticillus*, *Mesocestoides lineatus* (larva), and *Staphylepis cantaniana* [9]. Cestodes *Raillietina friedbergeri*, which we detected in a partridge in Poland for the first time, had previously been recorded only a few times in the world [34,35,36,37]. It was described for the first time in a pheasant in Germany [35,36], and later in partridges *Perdix perdix* in France [37] and the Ukraine [34]. The intermediate host in the life cycle of this tapeworm is the ant *Formica rufa* (Joyeux & Baer, 1937, after Kornyushin [34]), so birds become infected with it by eating ants. The pathogenicity of *R. friedbergeri* is unknown, and there is no reason to believe that infection with these tapeworms is harmful to birds. Trematodes (Digenea), previously recorded in partridges in Poland, are *Echinostoma revolutum* (Fröhlich, 1802) and *Prosthogonimus ovatus* (Rudolphi, 1803), so the detection of *Brachylaima* sp. in the material is a new addition to this list. In the Czech Republic, the species *B. fuscata* was recorded in the pheasant [2].

Previous attempts have been made to find a link between parasites and the poor survival rates of partridges in the environment. Brown et al. [7], for example, studied the relationship between the diet of partridges and susceptibility to parasitic diseases. They found that poor survival of wild broods is associated more with low habitat and food quality than with a high rate of infection by parasites. According to Bro et al. [51], predation is the main direct cause of mortality in females during breeding (73%) and the main factor determining survival rates. At the same time, the authors concluded that the direct effect of agricultural practices observed during the experiment was only 6% [51]. Kuijper et al. [14] conducted an in-depth analysis of the problem, investigating the causes of the dramatic decline in the numbers of partridges in Europe and suggesting the possibility of recovery. They concluded their review by stating that restoring the partridge population to the level from before 1950 could only take place as a result of large-scale habitat improvement [14].

### 4.3. Differences in Parasite Infection of Farm-Reared and Free-Living Partridges 

Our study showed that the infection of partridges with nematodes *C. phasianina* and *H. gallinarum* is associated with the way of life of birds; the prevalence of *C. phasianina* in partridges is higher in farmed flocks, whereas the prevalence of *Heterakis gallinarum* is higher in free-living partridges. This is most likely linked to the more frequent occurrence of invasive forms of *H. gallinarum* in nature, but it is difficult to say whether partridges were infected by *Capillaria* while still in rearing conditions or after being released into the wild. Kozakiewicz et al. [47] described differences in parasite infection of farmed and free-living partridges in Greater Poland. They showed that the prevalence of *Capillaria* sp. was 5% higher in partridges on the breeding farm (in the foundation stock) than in free-living partridges, and the prevalence of *Heterakis* was about 2% higher in farmed partridges. The authors explain that this is likely due to greater ‘contamination’ of the environment of farmed partridges with invasive forms of parasites, at the same time drawing attention to the high density of birds in aviaries. This can expose birds to contact with invasive forms of the nematode *Syngamus trachea* (Montagu, 1811), whose prevalence was similar to that of *Capillaria* sp. In our material from 2015–2017, no nematodes of the species *Ascaridia galli* or *Trichostrongylus tenuis* were found. The most recent reports of the occurrence of these two parasite species in partridges *Perdix perdix* date back to Kozakiewicz et al. [47]. 

## 5. Conclusions

In general, our findings indicate that parasites are more prevalent in pheasants than in partridges. The largest group of parasites in both species were nematodes, while cestodes and trematodes were less numerous. Pheasants are more often infected with intestinal nematodes and are a reservoir of these parasites in the environment. Nematodes, *Capillaria phasianina*, cestodes, *Raillietina friedbergeri*, and one trematode, *Brachylaima* sp. were recorded for the first time in partridges in Poland. The component community and infracommunity of parasites of partridges are more diverse and less dominated by a single parasite species than the pheasant parasite community. Given the high level of interest in the protection of partridges all over Europe, programmes for conservation of both partridges and pheasants should be supplemented with regular testing of the state of parasite infection of flocks of partridges and pheasants living in the wild. The results of parasitological examination should then be used to develop programmes for diagnosis and monitoring of parasitic infections in order to keep flocks free of parasites.

## Figures and Tables

**Table 1:** Partridges and pheasants from the Kuyavian-Pomeranian, Podlaskie, Silesian, and Świętokrzyskie Provinces, Poland, 2015–2017. Numbers indicate the number of game birds used in the analysis.

Host	Province	N*ex*	N*fr*	N*fa*	N*un*	Month
Grey partridge *Perdix perdix*	Świętokrzyskie	58	23	16	19	September and October
	Podlaskie	7	-	-	-	October
Common pheasant *Phasianus colchicus*	Silesian	27	-	27	-	February and December
	Kuyavian-Pomeranian	5	-	5	-	November

N*ex*, number of birds examined; N*fr*, number of free-living birds among tested birds; N*fa*, number of farm-reared birds among tested birds; N*un*, number of birds of unidentified origin among tested birds.

**Table 2:** Morphometric characteristics of nematodes *Capillaria phasianina.* Measurements are given in mm. Ranges of measurements reported by other authors are compared with our own results.

Parameter	Own Material							
	Male [32]	Female [32]	Male [4]	Female [4]	Male [33]	Female [33]	Male	Female
BL	14.7–19.0	22.0–28.0		28.91–32.36	14.0–26.5	20.9–37.3	14.15–18.6	19.02–22.95
BW-H	0.006–0.007	0.006–0.008					0.011	0.008–0.011
BW-V		0.065–0.084	13.9–20.22	0.060–0.070		0.040–0.075		0.30–0.056
BW-S					0.045–0.050		0.03–0.05	
V-BA				7–9		6.0–12.5		6.1–8.63
VA		0.070–0.082	0.052–0.070					0.056–0.075
CBW							0.047–0.056	
SL	1.55–2.40				1.770–2.660		1.781–2.04	
SW	0.017–0.027				0.023–0.031		0.017–0.028	
EL		0.052–0.057	1.9–2.25	0.050–0.055		0.046–0.060		0.056–0.061
EW		0.025–0.027		0.022–0.026		0.022–0.024		0.028–0.031
N.STICH	33–37	36–42					29–35	31–40

BL, body length; BW-H, body width at head; BW-V, body width at vulva; BW-S, body width at spicule height; V-BA, distance from vulval opening to body anterior; VA, length of vulval appendage; CBW width of copulatory bursa; SL, spicule length; SW, maximum spicule width; EL, egg length; EW, egg width; N.STICH, number of stichocytes.

**Table 3:** Ecological indices characterizing the structure of the component community and infracommunity and the prevalence and intensity of infection of partridges and pheasants, Poland, 2015–2017. The number of birds examined is given in brackets.

Host	Province	Parasites	N*ib*	P (%)	MI	RI
Grey partridge *Perdix perdix*	Świętokrzyskie (58)		29	50.0	13.4 ± 15.74	1–50
		*Raillietina friedbergeri*	6	10.3	8.3 ± 11.76	1–30
		*Brachylaima* sp.	1	1.7	1 ± 0.0	1–1
		*Capillaria phasianina*	9	15.5	11.1 ± 15.77	1–50
		*Capillaria* sp.	11	19.0	18.9 ± 17.9	1–50
		*Heterakis gallinarum*	11	19.0 *	2.7 ± 3.1	1–11
	Podlaskie (7)		0	0.0	-	
Common pheasant *Phasianus colchicus*	Silesian (27)		19	70.4	17.4 ± 43.96	1–191
		*Capillaria phasianina*	2	7.4	1 ± 0.0	1–1
		*Capillaria* sp.	3	11.1	3.3 ± 2.1	1–5
		*Heterakis gallinarum*	15	55.6 *	21.3 ± 48.0	1–191
	Kuyavian-Pomeranian (5)		0	0.0		

N*ib*, Number of infected birds; P, prevalence; MI, mean intensity; RI, range of intensity, * statistically significant difference between prevalence of *H. gallinarum* in partridges and in pheasants at *p* ≤ 0.05.

## Data Availability

The raw data are available from the authors.
